# New species of *Hymenogaster* (Agaricales, Hymenogastraceae) from North China

**DOI:** 10.3897/mycokeys.132.176986

**Published:** 2026-05-12

**Authors:** Ning Mao, Ting Li, Hao-Yu Fu, Yu-Xin Zhang, Yong-Jie Zhang, Li Fan

**Affiliations:** 1 School of Life Science, Shanxi University, Taiyuan 030006, China College of Life Science, Capital Normal University Beijing China; 2 Department of Life Sciences, National Natural History Museum of China, Tianqiaonandajie 126, Beijing 100050, China School of Life Science, Shanxi University Taiyuan China; 3 College of Life Science, Capital Normal University, Xisanhuanbeilu 105, Haidian, Beijing 100048, China Department of Life Sciences, National Natural History Museum of China Beijing China

**Keywords:** False-truffle, molecular analysis, new taxa, taxonomy

## Abstract

The genus *Hymenogaster* has significant ecological value and serves as a food source for numerous small mammals. Although numerous species have been reported from China, most lack confirmation by molecular evidence. In this study, six species of *Hymenogaster* were newly reported in China based on morphological and molecular phylogenetic analyses, five of which are new to science, and one of which is new to China. The descriptions and illustrations for new species are provided.

## Introduction

The Hymenogastraceae is a highly diverse fungal family that includes species with both agaricoid and false-truffle morphologies. Many members form ectomycorrhizal associations with trees across families such as Betulaceae, Fagaceae, and Pinaceae (Beker et al. 2016; Li et al. 2024a, 2024b). According to Hyde et al. (2024), the family currently encompasses 12 genera and approximately 1500 species. However, recent phylogenomic studies indicate that the Hymenogastraceae is not monophyletic, indicating that its current taxonomic framework requires revision (He et al. 2024).

*Hymenogaster* is the species-rich genus of the false truffles. This genus has a geographic pattern of global distribution across Asia, Europe, North America, Oceania, South America, and Africa (Harkness 1899; Dodge and Zeller 1934; Smith 1966; Pegler and Young 1987; Cázares and Trappe 1990; Montecchi et al. 2000; Fogel and States 2001; Julou et al. 2005; Trappe et al. 2009; Stielow et al. 2011; Pietras et al. 2013; Li et al. 2024a, 2024b). The key to distinguishing species morphologically hinges on the colour and discoloration of fresh basidiocarps and the morphology of the basidiospores (Dodge and Zeller 1934; Smith 1966; Cázares and Trappe 1990; Stielow et al. 2011; Li et al. 2024a, 2024b). According to the Index Fungorum database, the genus currently comprises 195 recorded names, of which approximately 75 are recognized as valid species (Hyde et al. 2024). In China, 39 species and one variety of *Hymenogaster* have been documented (Liu et al. 2002; Li et al. 2024a, 2024b). However, molecular data are available for only nine of these (Li et al. 2024a, 2024b). Consequently, the taxonomic status of most Chinese *Hymenogaster* species requires reassessment using molecular evidence.

During an investigation of *Hymenogaster* fungi in Shanxi Province of northern China, fruit bodies of *Hymenogaster* were collected. Morphological and molecular analyses revealed six species, including five new to science and one new to China of *Hymenogaster*. This study aims to enhance the taxonomic understanding of *Hymenogaster* by providing descriptions and illustrations of new species and supplying DNA sequence data for known species from China.

## Materials and methods

### Morphological Studies

Voucher specimens were accessioned in the Herbarium Biology Department at Capital Normal University (**BJTC**). Macroscopic characters were described from fresh and dried material. The microscopic characteristics’ observations of *Hymenogaster* were mainly based on Li et al. (2024a, 2024b).

### Molecular methods

Genomic DNA was extracted from dried basidiomes. ITS and LSU sequences were generated following methods detailed in White et al. (1990) and Li et al. (2024a, 2024b). Sequencing was carried out at Beijing Zhongkexilin Biotechnology Co. Ltd. (Beijing, China). Validated sequences are stored in the NCBI database under the accession numbers provided (Table 1).

**Table 1.** Sources of specimens and GenBank accession numbers for sequences used in this study. Newly generated sequences are in bold.

Taxon Name	Voucher Specimen	Country	ITS	nrLSU
*Hebeloma lactariolens*	taxon:301353	-	AY818352	-
*Hebeloma lactariolens*	HC 88/95	-	NR_119524	-
***Hymenogaster albo-ovoideus***	**BJTC FAN703**	**China**	**PZ288278**	**PZ288334**
***Hymenogaster albo-ovoideus***	**BJTC FAN1135**	**China**	**PZ288279**	**PZ288335**
***Hymenogaster albo-ovoideus***	**BJTC FAN1139**	**China**	**PZ288280**	**PZ288336**
***Hymenogaster albo-ovoideus***	**BJTC FAN1142**	**China**	**PZ288281**	**PZ288337**
***Hymenogaster albo-ovoideus***	**BJTC FAN1143**	**China**	**PZ288282**	**PZ288338**
***Hymenogaster albo-ovoideus***	**BJTC FAN1144**	**China**	**PZ288283**	**PZ288339**
*Hymenogaster arenarius*	BJTC FAN786	China	PP467413	PP467449
*Hymenogaster arenarius*	BJTC FAN856	China	PP467414	PP467450
*Hymenogaster arenarius*	it10_26_2	Germany	GU479233	-
*Hymenogaster arenarius*	it5_2	Germany	GU479272	-
*Hymenogaster arenarius*	it6_3	Germany	GU479278	-
*Hymenogaster bulliardii*	it20_4	Germany	GU479261	-
*Hymenogaster bulliardii*	it5_21	Germany	GU479273	-
*Hymenogaster bulliardii*	zb1485	Hungary	GU479308	-
*Hymenogaster citrinus*	BJTC FAN1079	China	PP467412	PP467448
*Hymenogaster citrinus*	BJTC FAN883	China	PP467410	PP467446
*Hymenogaster citrinus*	BJTC FAN915	China	PP467411	PP467447
*Hymenogaster citrinus*	dt8293	Belgium	GU479292	-
*Hymenogaster citrinus*	zb1645	Hungary	GU479313	-
*Hymenogaster citrinus*	zb1817	Hungary	GU479317	-
*Hymenogaster citrinus*	zb2300	Hungary	GU479332	-
*Hymenogaster gardneri*	Trappe 13296	USA	AF325638	-
*Hymenogaster gardneri*	SOC1643	USA	JN022510	-
*Hymenogaster gardneri*	Trappe 22752	USA	AF325640	-
*Hymenogaster griseus*	it16_1_1	Germany	GU479252	-
*Hymenogaster griseus*	aszodvt_1991	Hungary	GU479289	-
*Hymenogaster griseus*	zb1436	Hungary	GU479304	-
*Hymenogaster griseus*	zb2576	Hungary	GU479340	-
*Hymenogaster griseus*	Trappe 12841	USA	AF325636	-
*Hymenogaster griseus*	zb37	Hungary	GU479358	-
*Hymenogaster griseus*	zb3533	Hungary	GU479353	-
*Hymenogaster griseus*	zb2097	Hungary	GU479327	-
*Hymenogaster griseus*	zb2105	Hungary	GU479328	-
*Hymenogaster griseus*	zb3594	Hungary	GU479356	-
*Hymenogaster griseus*	17022	Italy	JF908082	-
*Hymenogaster huthii*	it12_3_1	Germany	GU479242	-
*Hymenogaster huthii*	zb95	Hungary	GU479366	-
*Hymenogaster intermedius*	it16_2, holotype	Germany	GU479253	-
***Hymenogaster intermedius***	**BJTC FAN552**	**China**	**PZ288318**	**PZ288372**
***Hymenogaster intermedius***	**BJTC FAN556**	**China**	**PZ288319**	**PZ288373**
***Hymenogaster intermedius***	**BJTC FAN558**	**China**	**PZ288320**	**PZ288374**
***Hymenogaster intermedius***	**BJTC FAN559**	**China**	**PZ288321**	**PZ288375**
***Hymenogaster intermedius***	**BJTC FAN562**	**China**	**PZ288322**	**PZ288376**
***Hymenogaster intermedius***	**BJTC FAN568**	**China**	**PZ288323**	**PZ288377**
***Hymenogaster intermedius***	**BJTC FAN577**	**China**	**PZ288324**	**PZ288378**
***Hymenogaster intermedius***	**BJTC FAN579**	**China**	**PZ288325**	**PZ288379**
***Hymenogaster intermedius***	**BJTC FAN581**	**China**	**PZ288326**	**-**
***Hymenogaster intermedius***	**BJTC FAN582**	**China**	**PZ288327**	**PZ288380**
***Hymenogaster intermedius***	**BJTC FAN594**	**China**	**PZ288328**	**PZ288381**
***Hymenogaster intermedius***	**BJTC FAN612**	**China**	**PZ288329**	**PZ288382**
***Hymenogaster intermedius***	**BJTC FAN619**	**China**	**PZ288330**	**PZ288383**
***Hymenogaster intermedius***	**BJTC FAN624**	**China**	**PZ288331**	**PZ288384**
***Hymenogaster intermedius***	**BJTC FAN665**	**China**	**PZ288332**	**PZ288385**
***Hymenogaster intermedius***	**BJTC FAN673**	**China**	**PZ288333**	**PZ288386**
*Hymenogaster latisporus*	KR-M-0044217	Germany	MT005942	-
*Hymenogaster latisporus*	BJTC FAN1134	China	PP467404	PP467440
*Hymenogaster latisporus*	zb1461	Hungary	GU479307	-
*Hymenogaster latisporus*	SC14_3	USA	KU878613	-
*Hymenogaster luteus*	zb1457	Hungary	GU479306	-
*Hymenogaster luteus*	zb2603	Hungary	GU479341	-
*Hymenogaster luteus*	zb235	Hungary	GU479334	-
*Hymenogaster luteus*	zb3721	Hungary	GU479359	-
*Hymenogaster megasporus*	it12_1	Germany	GU479239	-
*Hymenogaster megasporus*	it8_5_1	Germany	GU479286	-
*Hymenogaster minisporus*	BJTC FAN1244	China	PP467407	PP467443
*Hymenogaster minisporus*	QL054	China	HM105539	-
*Hymenogaster minisporus*	taxon:522720	China	LT980461	-
*Hymenogaster niveus*	it17_3	Germany	GU479255	-
*Hymenogaster niveus*	FV4_04	Slovenia	MK027200	-
*Hymenogaster niveus*	KR-M-0044314	Germany	MT005967	-
*Hymenogaster niveus*	zb28	Hungary	GU479344	-
“*Hymenogaster niveus*”	KR-M-0044314	Germany	MT005967	-
“*Hymenogaster niveus*”	RBG Kew K(M)64578	_	EU784364	-
“*Hymenogaster niveus*”	Trappe 599	_	AF325632	-
*Hymenogaster papilliformis*	BJTC FAN1002	China	PP467396	PP467432
*Hymenogaster papilliformis*	BJTC FAN1070	China	PP467399	PP467435
*Hymenogaster papilliformis*	BJTC FAN1074	China	PP467400	PP467436
*Hymenogaster papilliformis*	BJTC FAN1109	China	PP467402	PP467438
*Hymenogaster papilliformis*	BJTC FAN1156	China	PP467406	PP467442
*Hymenogaster papilliformis*	BJTC FAN1266	China	PP467408	PP467444
*Hymenogaster papilliformis*	BJTC FAN1267	China	PP467409	PP467445
*Hymenogaster papilliformis*	BJTC FAN655	China	PP467381	PP467417
*Hymenogaster papilliformis*	BJTC FAN807	China	PP467384	PP467420
*Hymenogaster papilliformis*	BJTC FAN820	China	PP467385	PP467421
*Hymenogaster papilliformis*	BJTC FAN891	China	PP467388	PP467424
*Hymenogaster papilliformis*	BJTC FAN944	China	PP467389	PP467425
*Hymenogaster papilliformis*	BJTC FAN958	China	PP467391	PP467427
*Hymenogaster papilliformis*	BJTC FAN960	China	PP467392	PP467428
*Hymenogaster papilliformis*	BJTC FAN980	China	PP467393	PP467429
*Hymenogaster papilliformis*	BJTC FAN983	China	PP467394	PP467430
*Hymenogaster papilliformis*	BJTC FAN992	China	PP467395	PP467431
*Hymenogaster perisporius*	BJTC FAN1038	China	PP467397	PP467433
*Hymenogaster perisporius*	BJTC FAN1049	China	PP467398	PP467434
*Hymenogaster perisporius*	BJTC FAN1076	China	PP467401	PP467437
*Hymenogaster perisporius*	BJTC FAN1126	China	PP467403	PP467439
*Hymenogaster perisporius*	BJTC FAN606	China	PP467379	PP467415
*Hymenogaster perisporius*	BJTC FAN651	China	PP467380	PP467416
*Hymenogaster perisporius*	BJTC FAN768	China	PP467383	PP467419
*Hymenogaster perisporius*	BJTC FAN846	China	PP467386	PP467422
*Hymenogaster perisporius*	BJTC FAN850	China	PP467387	PP467423
*Hymenogaster perisporius*	BJTC FAN952	China	PP467390	PP467426
***Hymenogaster pinophilus***	**BJTC FAN560**	**China**	**PZ288307**	**PZ288362**
***Hymenogaster pinophilus***	**BJTC FAN565**	**China**	**PZ288308**	**PZ288363**
***Hymenogaster pinophilus***	**BJTC FAN571**	**China**	**PZ288309**	**PZ288364**
***Hymenogaster pinophilus***	**BJTC FAN583**	**China**	**PZ288310**	**PZ288365**
***Hymenogaster pinophilus***	**BJTC FAN602**	**China**	**PZ288311**	**PZ288366**
***Hymenogaster pinophilus***	**BJTC FAN607**	**China**	**PZ288312**	**PZ288367**
***Hymenogaster pinophilus***	**BJTC FAN668**	**China**	**PZ288313**	**PZ288368**
***Hymenogaster pinophilus***	**BJTC FAN678**	**China**	**PZ288314**	**-**
***Hymenogaster pinophilus***	**BJTC FAN685**	**China**	**PZ288315**	**PZ288369**
***Hymenogaster pinophilus***	**BJTC FAN687**	**China**	**PZ288316**	**PZ288370**
***Hymenogaster pinophilus***	**BJTC FAN702**	**China**	**PZ288317**	**PZ288371**
*Hymenogaster pseudoniveus*	BJTC FAN874	China	PP622380	PP622367
*Hymenogaster pseudoniveus*	BJTC FAN916	China	PP622383	PP622370
*Hymenogaster pseudoniveus*	BJTC FAN967	China	PP622382	PP622369
*Hymenogaster pseudoniveus*	BJTC FAN1069	China	PP622379	PP622366
*Hymenogaster pseudoniveus*	BJTC FAN1075	China	PP622378	PP622365
*Hymenogaster pseudoniveus*	BJTC FAN1238	China	PP622384	-
*Hymenogaster pseudoniveus*	BJTC FAN1251	China	PP622381	PP622368
*Hymenogaster rehsteineri*	KR-M-0044423	Germany	MT005990	-
*Hymenogaster rehsteineri*	it2_4_1	Germany	GU479259	-
*Hymenogaster rehsteineri*	dt8455	Luxembourg	GU479293	-
*Hymenogaster rehsteineri*	KR-M-0044018	Germany	MT005953	-
*Hymenogaster rehsteineri*	RBG Kew K(M)27363	-	EU784365	-
*Hymenogaster rehsteineri*	GN_4d_I	Sweden	JQ724028	-
“*Hymenogaster rehsteineri*”	AF91	Australia	DQ328132	-
“*Hymenogaster rehsteineri*”	zb483	Hungary	GU479363	-
“*Hymenogaster rehsteineri*”	it3_4_1	Germany	GU479266	-
“*Hymenogaster rehsteineri*”	951_PREMIX	USA	OQ612522	-
“*Hymenogaster rehsteineri*”	HMJAU65151	China	OQ750231	-
“*Hymenogaster rehsteineri*”	KR-M-0044347	Germany	MT005946	-
*Hymenogaster* sp.	GP 5302	-	AF325634	-
*Hymenogaster* sp.	Fogel 2698	USA	AY945303	-
*Hymenogaster* sp.	A2N_88	Canada	EU554705	-
*Hymenogaster* sp.	A3E_60	Canada	EU554717	-
*Hymenogaster* sp.	HAY-F-003960	USA	OR858776	-
*Hymenogaster* sp.	FLAS-F-69843	USA	OQ150515	-
*Hymenogaster* sp.	L5D5_2	USA	JX030266	-
*Hymenogaster* sp.	HAD1518	USA	PV832886	-
*Hymenogaster* sp.	L2LvC	USA	JX030221	-
*Hymenogaster* sp.	LM3228	France	KM576608	-
*Hymenogaster* sp.	O2Lv_3_11	USA	JX030281	-
*Hymenogaster* sp.	JLF13650	USA	PQ523741	-
***Hymenogaster subluteus***	**BJTC FAN1087**	**China**	**PZ288303**	**-**
***Hymenogaster subluteus***	**BJTC FAN1094**	**China**	**PZ288304**	**PZ288359**
***Hymenogaster subluteus***	**BJTC FAN1104**	**China**	**PZ288305**	**PZ288360**
***Hymenogaster subluteus***	**BJTC FAN1113**	**China**	**PZ288306**	**PZ288361**
*Hymenogaster tener*	RBG Kew K(M)102406	UK	EU784363	-
*Hymenogaster tener*	it15_3	Germany	GU479250	-
***Hymenogaster tenuihymenium***	**BJTC FAN847**	**China**	**PZ288284**	**PZ288340**
***Hymenogaster thermophilus***	**BJTC FAN942**	**China**	**PZ288285**	**PZ288341**
***Hymenogaster thermophilus***	**BJTC FAN1089**	**China**	**PZ288286**	**PZ288342**
***Hymenogaster thermophilus***	**BJTC FAN1090**	**China**	**PZ288287**	**PZ288343**
***Hymenogaster thermophilus***	**BJTC FAN1096**	**China**	**PZ288288**	**PZ288344**
***Hymenogaster thermophilus***	**BJTC FAN1099**	**China**	**PZ288289**	**PZ288345**
***Hymenogaster thermophilus***	**BJTC FAN1114**	**China**	**PZ288290**	**PZ288346**
***Hymenogaster thermophilus***	**BJTC FAN1115**	**China**	**PZ288291**	**PZ288347**
***Hymenogaster thermophilus***	**BJTC FAN1122**	**China**	**PZ288292**	**PZ288348**
***Hymenogaster thermophilus***	**BJTC FAN1125**	**China**	**PZ288293**	**PZ288349**
***Hymenogaster thermophilus***	**BJTC FAN1129**	**China**	**PZ288294**	**PZ288350**
***Hymenogaster thermophilus***	**BJTC FAN1130**	**China**	**PZ288295**	**PZ288351**
***Hymenogaster thermophilus***	**BJTC FAN1132**	**China**	**PZ288296**	**PZ288352**
***Hymenogaster thermophilus***	**BJTC FAN1147**	**China**	**PZ288297**	**PZ288353**
***Hymenogaster thermophilus***	**BJTC FAN1159**	**China**	**PZ288298**	**PZ288354**
***Hymenogaster thermophilus***	**BJTC FAN1257**	**China**	**PZ288299**	**PZ288355**
***Hymenogaster thermophilus***	**BJTC FAN1265**	**China**	**PZ288300**	**PZ288356**
***Hymenogaster thermophilus***	**BJTC FAN1269**	**China**	**PZ288301**	**PZ288357**
***Hymenogaster thermophilus***	**BJTC FAN1270**	**China**	**PZ288302**	**PZ288358**
*Hymenogaster thwaitesii*	it20_4_1	Germany	GU479262	-
*Hymenogaster thwaitesii*	it9_2	Germany	GU479287	-
*Hymenogaster thwaitesii*	zb2804	Hungary	GU479345	-
*Hymenogaster thwaitesii*	it2_2	Germany	GU479258	-
*Hymenogaster thwaitesii*	it3_2	Germany	GU479264	-
*Hymenogaster variabilis*	BJTC FAN1141	China	PP467405	PP467441
“*Hymenogaster vulgaris*”	FS3	Slovakia	MT929774	-
*Hymenogaster variabilis*	BJTC FAN656	China	PP467382	PP467418
*Hymenogaster zunhuaensis*	BJTC FAN1061	China	PP622373	PP622361
*Hymenogaster zunhuaensis*	BJTC FAN1062	China	PP622376	PP622363
*Hymenogaster zunhuaensis*	BJTC FAN1083	China	PP622374	PP622362
*Hymenogaster zunhuaensis*	BJTC FAN1105	China	PP622372	PP622360
*Hymenogaster zunhuaensis*	BJTC FAN1162	China	PP622375	-
*Hymenogaster zunhuaensis*	BJTC FAN1249	China	PP622377	PP622364
*Hymenogaster zunhuaensis*	BJTC FAN1259	China	PP622371	PP622359
Uncultured ectomycorrhizal fungus		USA	AY310842	-
Uncultured fungus	hozan2022_557	Japan	LC711550	-
Uncultured fungus	RO-K2_E07	Switzerland	KX886027	-
Uncultured *Hymenogaster*	H07IK19	Germany	HF675446	-
Uncultured *Hymenogaster*	H07IK09	Germany	HF675437	-
Uncultured *Hymenogaster*	ectomycorrhiza7	-	ON454513	-
Uncultured *Hymenogaster*	ectomycorrhiza23	-	ON454529	-

A concatenated ITS-LSU dataset was used to investigate the phylogenetic placement of the *Hymenogaster* species. *Hebeloma* species were selected as the outgroup. The sequences of each marker were independently aligned in MAFFT (Katoh and Standley 2013) under default parameters, and manually adjusted for maximum sequence similarity in Se-Al v. 2.03a (Rambaut 2000). Ambiguously aligned regions and gaps in the alignment were excluded before the analyses. For the concatenated dataset, alignments were constructed separately for each of the gene fragments using MAFFT (Katoh and Standley 2013), optimized using BioEdit v. 7.0.9 (Hall 1999), then concatenated using SequenceMatrix v. 1.7.8 (Vaidya et al. 2011). Maximum Likelihood (ML) and Bayesian Inference (BI) analyses were conducted on the resulting concatenated dataset. Maximum likelihood (ML) analyses on datasets in this study were conducted with RAxML v. 8.0.14 (Stamatakis 2014) and the GTRGAMMAI substitution model with parameters unlinked. The ML bootstrap replicates (1000) were computed in RAxML with a rapid bootstrap analysis and search for the best-scoring ML tree. Bayesian inference (BI) was performed with MrBayes v. 3.2.2 (Ronquist and Huelsenbeck 2003). The best substitution model that fit the data at each locus was evaluated using MrModeltest v. 2.3 (Nylander 2004). For the concatenated analyses, each locus was considered a partition and assigned its own best-fitting substitution model, that was GTR+I+G for all partitions. We used two independent runs with four Markov chains Monte Carlo (MCMC) for 5 155 000 generations under the default settings. Average standard deviations of split frequency (ASDSF) values were far lower than 0.01 at the end of the generations. Trees were sampled every 100 generations after burn-in (25% of trees were discarded as the burn-in phase of the analyses, set up well after convergence), and 50% majority-rule consensus trees were constructed.

Clades with bootstrap support (BS) ≥ 70% and Bayesian posterior probability (PP) ≥ 0.95 were considered as significantly supported (Hillis and Bull 1993; Alfaro et al. 2003). All phylogenetic trees were viewed with TreeView (Page 2001).

## Results

### Phylogenetic analyses

**Figure 1.**
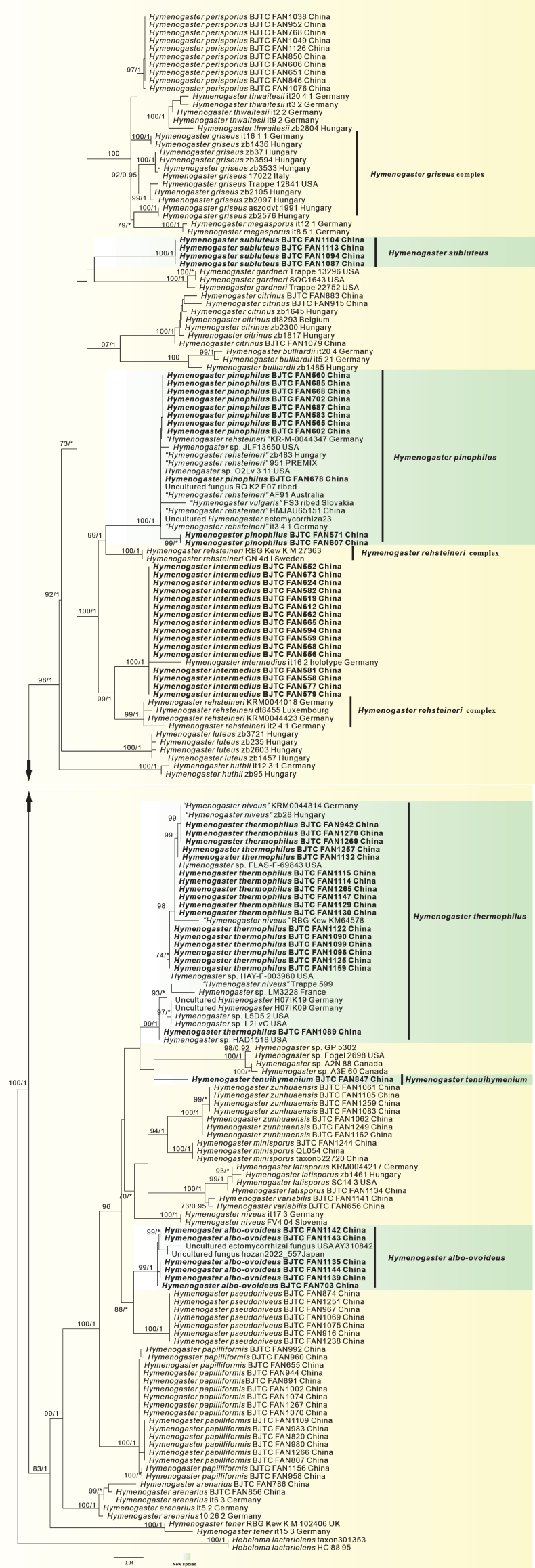
Phylogeny derived from maximum likelihood analyses of the ITS/LSU sequences from *Hymenogaster* and related species. Values in the left represent likelihood bootstrap support values (≥ 70%). Values in the right represent significant Bayesian posterior probability values (≥ 95%). Novel sequences are in bold.

The concatenated dataset (ITS/LSU) contained 291 sequences from 190 samples, including 109 sequences newly generated from our collections. The length of the aligned dataset was 1 494 bp after exclusion of poorly aligned sites (623 bp for ITS and 871 bp for LSU). The topologies of ML and BI phylogenetic trees obtained in this study were practically the same, therefore only the tree inferred from the ML analysis is shown (Fig. 1). Our phylogenetic analyses confirmed the monophyly of *Hymenogaster*. The sequences from our specimens clustered into six well-supported clades, supporting the recognition of six separate species (see Taxonomy in this paper). Five of these clades are newly proposed species, while the remaining one corresponds to a known species, *H. intermedius* Stielow, Bratek & Hensel, which is new to China.

### Taxonomy

#### Hymenogaster albo-ovoideus

Taxon classificationFungiAgaricalesHymenogastraceae

L. Fan, T. Li & N. Mao sp. nov.

969A8637-14AE-5484-9EFB-2903769A9208

863511

Fig. 2

##### Etymology.

*albo-ovoideus* referring to the combination of the snow-white basidiomes and the broadly ovoid to ovoid basidiospores.

##### Holotype.

China • Shanxi Province, Yuncheng City, Yuanqu County, Lishan Town, Shunwangping, alt. 1744 m, in the soil under *Betula costata*, 30 October 2017, LT069 (BJTC FAN1139).

##### Description.

***Basidiomes*** hypogeous, subglobose to irregularly globose, 0.5–1 cm diam, soft and elastic, snow white or white when fresh, turning yellowish white to yellowish brown, sometimes dark brown when dry. Surface smooth, glabrous, with a distinct depression at the sterile base. ***Peridium*** 110–360 μm thick, pseudoparenchymatous, composed of ellipsoid to subglobose cells, 8–15 μm in diam, and interwoven hyphae, hyphae hyaline to pale yellow-brown, 1.3–1.6 μm broad, not inflated. ***Gleba*** white when young, turning yellowish brown to brown at maturity, loculate, locules irregular in length, empty, filled with spores at maturity. ***Hymenium*** 21–49 μm thick. ***Basidia*** clavate, 1–4-spored, up to 10 μm over than hymenium, sterigmata 3–7 μm long, collapsed and disappeared at maturity.

**Figure 2.**
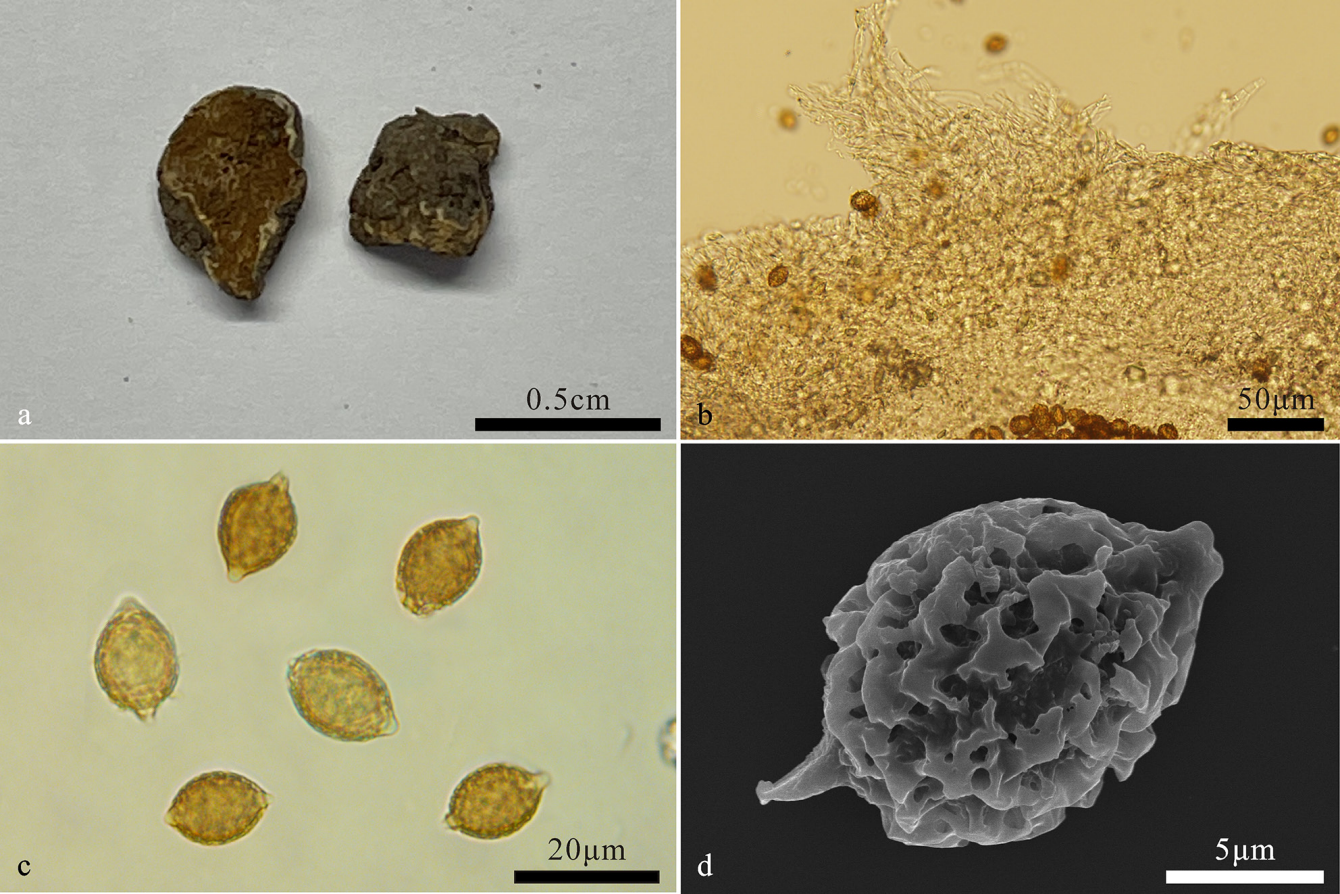
*Hymenogaster albo-ovoideus* (BJTC FAN1139, holotype). **a**. Basidiomes; **b**. Peridium; **c**. Basidiospores; **d**. Basidiospore under SEM.

***Basidiospores*** broadly ovoid to ovoid, pale yellowish brown to yellowish brown at maturity, ornamented with densely verrucose and ridges of 1–2 μm high, ridges short, irregular, interwoven, excluding ornamentations, 11.8–14.2(–15) × 9.9–12 μm (Lm × Wm = 12.9 ± 0.8 μm × 10.8 ± 0.5 μm, n = 30), Q = 1.2–1.3 (Q_av_ = 1.2 ± 0.03, n = 30), gelatinous perisporium present, with an apical hump, obtuse, hyaline, 1–2(–3) μm high, with a pronounced basal sterigma, truncate, 1–2 μm long.

##### Habit.

Hypogeous, in the soil under *Betula costata*, Shanxi Province, northern China.

##### Additional specimens examined.

China • Shanxi Province, Yuncheng City, Yuanqu County, Lishan Town, Shunwangping, alt. 2179 m, 17 October 2016, in soil under *Betula costata*, YXY034 (BJTC FAN703); • ibid., alt. 1744 m, 30 October 2017, YXY150 (BJTC FAN1135), LT072 (BJTC FAN1142), LT073 (BJTC FAN1143); • ibid., Jincheng City, Yangcheng County, Manghe Nature Reserve, alt. 580 m, XYY081 (BJTC FAN1144).

##### Notes.

*Hymenogaster albo-ovoideus* is characterized by snow white to white basidiomes, broadly ovoid to ovoid basidiospores with densely verrucose and ridge-like ornamentations. *Hymenogaster albo-ovoideus* is easily confused with *H. niveus* Vittad. by their similar morphology with snow white basidiomes (Dodge and Zeller 1934; Stielow et al. 2011). However, *H. niveus* differs from *H. albo-ovoideus* by its basidiomes turning red upon bruising or handling and diverse basidiospore ornamentations (Stielow et al. 2011; Dodge and Zeller 1934). *Hymenogaster albo-ovoideus* was phylogenetically sister to *H. pseudoniveus* L. Fan & Ting Li, a new species found in Hebei, Shanxi and Shaanxi Provinces, Northern China. However, *H. pseudoniveus* differs from *H. albo-ovoideus* by its ellipsoid to broadly ellipsoid basidiospores (Li et al. 2024b).

Two ITS sequences (LC711550, AY310842) extracted from ectomycorrhizal root tips downloaded from GenBank matched *H. albo-ovoideus* in our analysis, one of which was from Japan (LC711550), and another from USA (AY310842). That could imply that *H. albo-ovoideus* may occur in both Japan and the USA. Moreover, BLASTn revealed that *H. albo-ovoideus* shares less than 95.75% ITS similarity with sequences from the NCBI database.

#### Hymenogaster pinophilus

Taxon classificationFungiAgaricalesHymenogastraceae

N. Mao, L. Fan & T. Li sp. nov.

BC6EFE93-3CE5-5299-B459-5563279C1C69

863512

Fig. 3

##### Etymology.

*pinophilus* (pine-loving) referring to the habitat of the species amongst forest of *Pinus*.

##### Holotype.

China • Shanxi Province, Yuncheng City, Yuanqu County, Lishan Town, Shunwangping, alt. 2209 m, in the soil under *Pinus armandii*, 16 August 2017, HBD008 (BJTC FAN583).

##### Description.

***Basidiomes*** hypogeous, subglobose to irregularly globose, 0.5–2 cm diam, soft and elastic, cream white to white when fresh, turning yellowish white to pale yellowish brown when dry. Surface smooth, glabrous, with a distinct depression at the sterile base. ***Peridium*** 87–150 μm thick, pseudoparenchymatous, composed of ellipsoid to subglobose cells, 7–17 μm in diam, and interwoven hyphae, hyphae pale yellow, 1.4–2.5 μm broad, not inflated. ***Gleba*** yellowish brown to black brown at maturity, loculate, locules irregular in length, empty, filled with spores at maturity. ***Hymenium*** 20–37 μm thick. ***Basidia*** clavate, 1–2-spored, mainly 2-spored, sterigmata 3–7 μm long, collapsed and disappeared at maturity.

**Figure 3.**
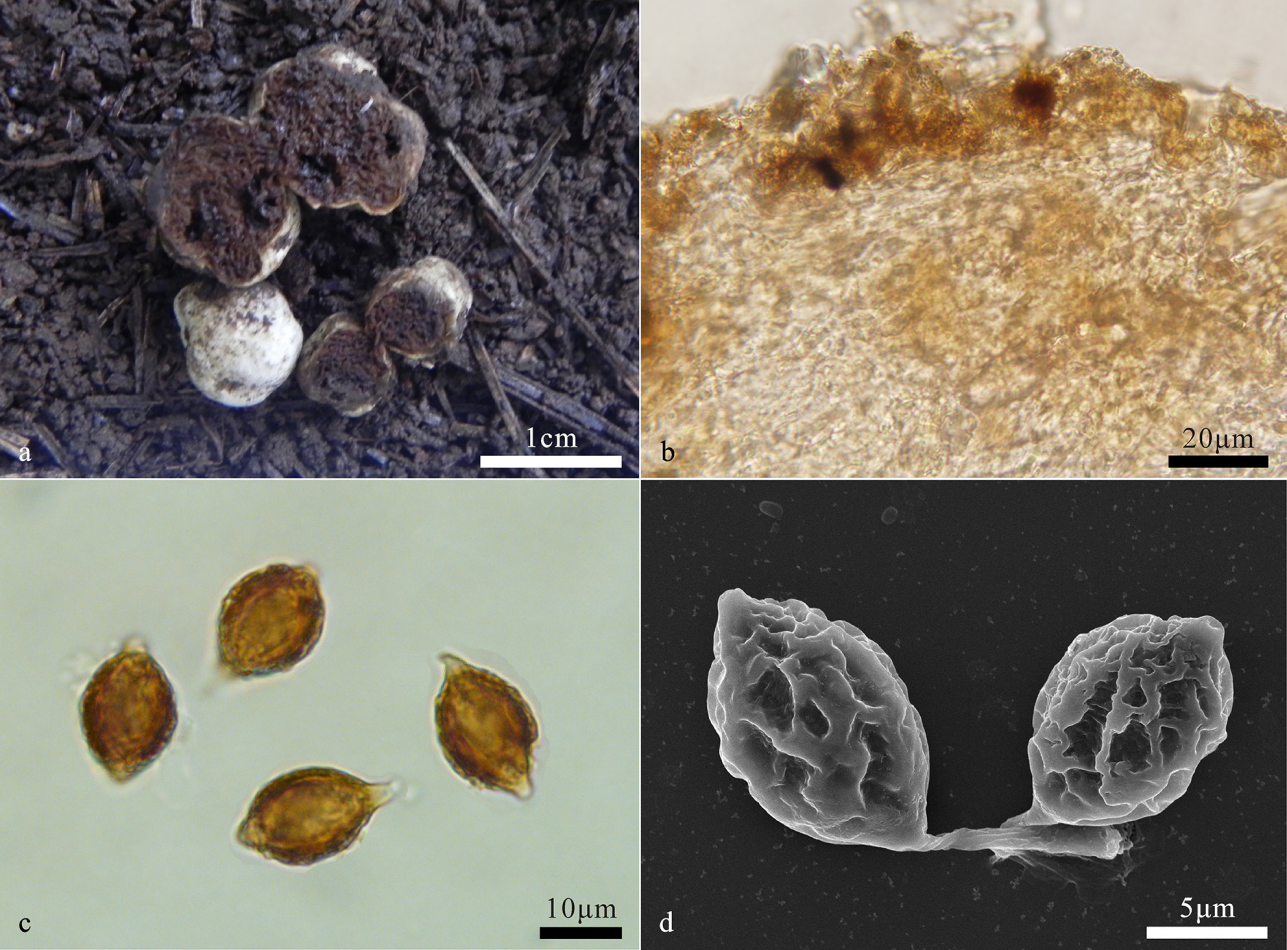
*Hymenogaster pinophilus* (BJTC FAN583, holotype). **a**. Basidiomes; **b**. Peridium; **c**. Basidiospores; **d**. Basidiospores under SEM.

***Basidiospores*** fusiform, broadly fusiform to ovoid, yellowish brown to brown at maturity, ornamented with densely ridges of 1–2 μm high, ridges short, irregular, interwoven, 14.5–18.8(–19.3) × 10–14.2 μm (Lm × Wm = 17.1 ± 1.5 × 11.8 ± 1.1 μm, n = 30), Q = 1.3–1.6 (Q_av_ = 1.45 ± 0.1, n = 30), gelatinous perisporium present, with a apical hump, obtuse, hyaline, 1–2(–3) μm high, with a pronounced basal sterigma, truncate, 2–3(–4) μm long.

##### Habit.

Hypogeous, in the soil under *Pinus armandii*, Shanxi Province, northern China.

##### Additional specimens examined.

China • Shanxi Province, Yuncheng City, Yuanqu County, Lishan Town, Shunwangping, alt. 2209 m, 16 August 2017, WYW008 (BJTC FAN560); • ibid., HKB016 (BJTC FAN565); • ibid., CM005 (BJTC FAN571); • ibid., YXY008 (BJTC FAN602); • ibid., YXY002 (BJTC FAN607); • ibid., alt. 2276 m, 17 October 2017, YXY036 (BJTC FAN668), • ibid., YXY 038 (BJTC FAN678), • ibid., YXY035 (BJTC FAN687); • ibid., SXY 017(BJTC FAN685); • ibid., CM 013(BJTC FAN702)

##### Notes.

*Hymenogaster pinophilus* is characterized by yellowish brown to black brown gleba, fusiform, broadly fusiform to ovoid basidiospores and habitat associated with *Pinus armandii*. BLASTn revealed that *H. pinophilus* shares less than 95.75% ITS similarity with sequences from the NCBI database.

11 sequences downloaded from NCBI well matched our *H. pinophilus* with strong support, indicating that the present species also occurs in Europe, North America, Asia and Australia, and has a wide range of host trees including *Castanea hybrid*, *Fagus sylvatica*, *Picea abies*, *Salix* sp. Those labeled as *H. rehsteineri* and *H. vulgaris* were actually misidentifications as the basidiospores in *H. rehsteineri* are shaped papillate to lanceolate, while the basidiospores in *H. vulgaris* are ornamented small warts. All of those are clearly different from the present species (Dodge and Zeller 1934; Stielow et al. 2011).

*Hymenogaster intermedius* is closely related to *H. pinophilus* and morphologically similar, but *H. intermedius* differs by its globose to subglobose basidiospores (Stielow et al. 2011; Dodge and Zeller 1934).

#### Hymenogaster subluteus

Taxon classificationFungiAgaricalesHymenogastraceae

N. Mao, L. Fan & T. Li sp. nov.

4CDCAB08-A3AD-5573-BC1F-131063326B1A

863513

Fig. 4

##### Etymology.

*subluteus*, referring to the similarity to *H. luteus* in appearance of basidiomes.

##### Holotype.

China • Shanxi Province, Yuncheng City, Xia County, alt. 970 m, 27 October 2017, in the soil under *Quercus variabilis*, YXY126 (BJTC FAN1094).

##### Description.

***Basidiomes*** hypogeous, depressed globose, subglobose to globose, 0.8–3 cm diam, soft and elastic, white, cream white, dirty white to pale yellow. Surface smooth, glabrous, with a distinct depression at the sterile base. ***Peridium*** 210–357 μm thick, pseudoparenchymatous, composed of ellipsoid to subglobose cells, 8–10 μm in diam, and interwoven hyphae, hyphae hyaline to pale yellow-brown, 2.5–6 μm broad, not inflated. ***Gleba*** reddish brown to ochre brown at maturity, loculate, locules irregular in length, empty, filled with spores at maturity. ***Hymenium*** 20–48 μm thick. ***Basidia*** clavate, 1–2-spored, up to 17–25 μm over than hymenium, sterigmata 2–4 μm long, collapsed and disappeared at maturity.

**Figure 4.**
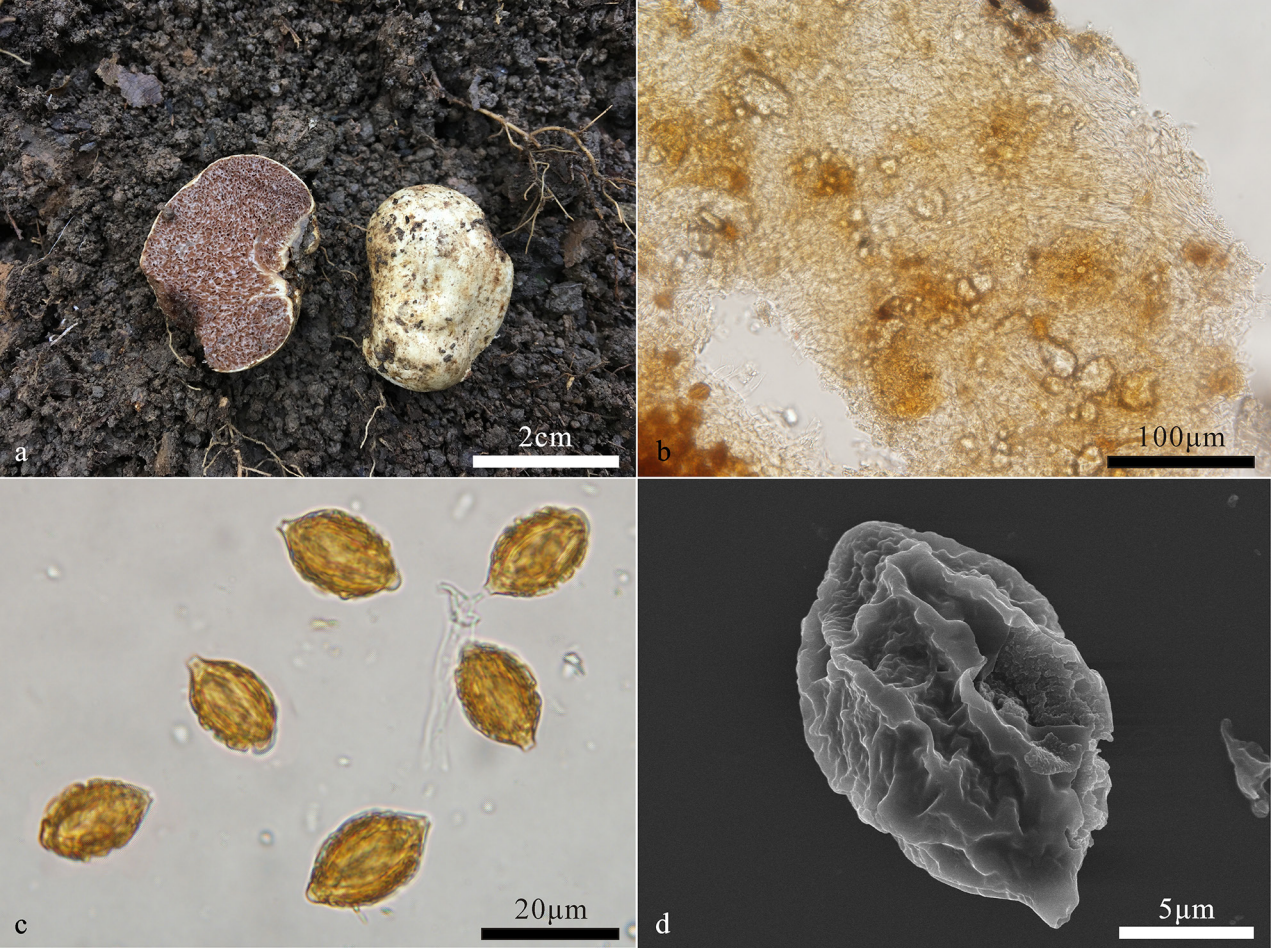
*Hymenogaster subluteus* (BJTC FAN1094, holotype). **a**. Basidiome; **b**. Peridium; **c**. Basidiospores; **d**. Basidiospore under SEM.

***Basidiospores*** fusiform, yellowish brown at maturity, ornamented with densely verrucose and ridges of 0.5–1 μm high, ridges short, irregular, interwoven, (13.2–)14.3–17.4(–18.3) × (9.3–)10.2–12.5(–13) μm (Lm × Wm = 15.9 ± 0.9 × 11.4 ± 0.6 μm, n = 30), Q = 1.2–1.5 (Q_av_ = 1.4 ± 0.07, n = 30), gelatinous perisporium present, with a apical hump, obtuse, hyaline, 1––2 μm high, with a pronounced basal sterigma, truncate, 1–2 μm long.

##### Habit.

Hypogeous, in the soil under *Quercus variabilis*, Shanxi Province, northern China.

##### Additional specimens examined.

China • Shanxi Province, Yuncheng City, Xia County, alt. 970 m, 27 October 2017, LT061 (BJTC FAN1087); XYY074 (BJTC FAN1104); • alt. 2003 m, 27 October 2017, HKB151 (BJTC FAN1113).

##### Notes.

*Hymenogaster subluteus* is characterized by white, cream white, dirty white to pale yellow basidiomes, fusiform basidiospores with densely verrucose and ridge-like ornamentations. *Hymenogaster luteus* Vittad is macroscopically similar to *H. subluteus* and both share white to yellow basidiomes, which makes them difficult to distinguish. However, they are easily separated by their basidiospore morphology, with larger [(18–22 (-28) × 9–11) μm], smooth basidiospores in *H. luteus* and smaller, ornamented basidiospores in *H. subluteus* (Dodge and Zeller 1934). *Hymenogaster gardneri* Zeller & Dodge is phylogenetically close to *H. subluteus*. However, *H. gardneri* differs from *H. subluteus* by its sordide albidae brunhescentes basidioma and broad-ellipsoid basidiospores (Dodge and Zeller 1934). Moreover, BLASTn revealed that *H. subluteus* shares less than 92.8% ITS similarity with sequences from the NCBI database.

#### Hymenogaster tenuihymeninus

Taxon classificationFungiAgaricalesHymenogastraceae

T. Li, L. Fan & N. Mao sp. nov.

D14CAA5F-9EA3-5E5F-AAD7-EA7A2BFB24E4

863514

Fig. 5

##### Etymology.

*tenuihymeninus* referring to the distinctly thin hymenium.

##### Holotype.

China • Shanxi Province, Lvliang City, Jiaocheng Country, Shenweigou, alt. 2003 m, in the soil under *Betula costata*, 7 September 2017, YXY060 (BJTC FAN847).

##### Description.

***Basidiome*** hypogeous, globose to subglobose, 0.5–0.6 cm diam, soft and elastic, white to pale yellow when fresh, turning pale brown when dry. Surface smooth, glabrous. ***Peridium*** 54–78 μm thick, pseudoparenchymatous, composed of subglobose cells, 8–17 μm in diam, and interwoven hyphae, hyphae hyaline, 2.5–4.5 μm broad, not inflated. ***Gleba*** white to pale yellow at maturity, turning pale brown when dry, loculate, locules irregular in length, empty, filled with spores at maturity. ***Hymenium*** 20–30 μm thick. ***Basidia*** clavate, 1–2-spored, up to 32–42 μm over than hymenium, sterigmata 2.5–3.5 μm long, collapsed and disappeared at maturity.

**Figure 5.**
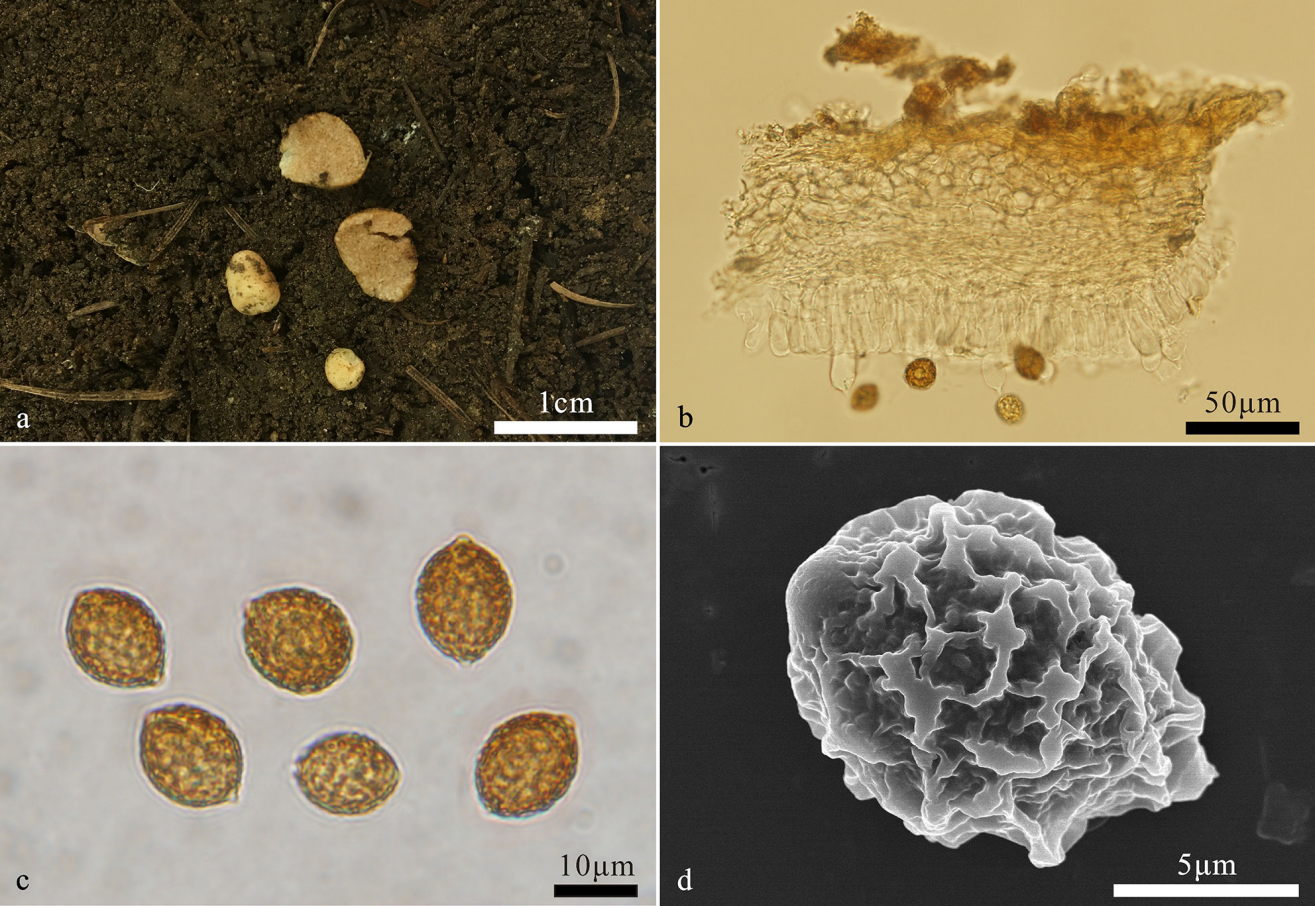
*Hymenogaster tenuihymeninus* (BJTC FAN847, holotype). **a**. Basidiomes; **b**. Peridium; **c**. Basidiospores; **d**. Basidiospore under SEM.

***Basidiospores*** broadly ellipsoid to subglobose, yellowish brown at maturity, ornamented with densely verrucose and ridges of 1 μm high, ridges short, irregular, interwoven, excluding ornamentations, (11.3–)12.3–14.1(–15) × (9.7–)10.3–12.1(–12.6) μm (Lm × Wm = 13.2 ± 0.5 μm × 11.3 ± 0.5 μm, n = 30), Q = 1.1–1.3 (Q_av_ = 1.2 ± 0.1, n = 30), gelatinous perisporium present, with an inconspicuous apical hump, hyaline, up to 1 μm high, with a pronounced basal sterigma, truncate, 1–2 μm long.

##### Habit.

Hypogeous, in the soil under *Betula costata*, Shanxi Province, northern China.

##### Notes.

*Hymenogaster tenuihymeninus* is characterized by distinctly thin hymenium, broadly ellipsoid to subglobose basidiospores with an inconspicuous apical hump. Phylogenetically, it is closely to *H. thermophilus*. Both species share a white to yellowish-white basidiomes coloration. However, *H. thermophilus* can be distinguished from *H. tenuihymeninus* by its basidiospores with a distinct apical hump and a habitat primarily associated with *Quercus* forests. *Hymenogaster zunhuaensis* L. Fan & Ting Li, a species recently discovered in Shanxi, is phylogenetically closely related to *H. tenuihymeninus*. However, it can be easily distinguished from *H tenuihymenium* by its gleba, which exhibits reddish to rusty tinges, and a relatively thick hymenium (up to 55 µm) (Li et al. 2024b). Moreover, BLASTn revealed that *H. tenuihymeninus* shares less than 94.6% ITS similarity with sequences from the NCBI database.

#### Hymenogaster thermophilus

Taxon classificationFungiAgaricalesHymenogastraceae

T. Li, L. Fan & N. Mao sp. nov.

DBDC8D31-8F5A-53E8-A99B-B60401FAB867

863515

Fig. 6

##### Etymology.

*thermophilus* referring to the species preferred to grow in the relatively warm climate regions of Shanxi Province.

##### Holotype.

China • Shanxi Province, Yuncheng City, Xia County, alt. 978 m, in the soil under *Quercus* sp.,7 October 2020, LT154 (BJTC FAN1269).

##### Description.

***Basidiomes*** hypogeous, globose, oblate to ellipsoid, 1–1.5 cm diam, soft and elastic, white to yellowish white when fresh, turning dirty white to pale yellow when dry. Surface smooth, glabrous, with a distinct depression at the sterile base. ***Peridium*** 105–220 μm thick, pseudoparenchymatous, composed of ellipsoid to subglobose cells, 7–8 μm in diam, and interwoven hyphae, hyphae hyaline to pale yellow-brown, 1.2–2.5 μm broad, not inflated. ***Gleba*** pale yellow at maturity, turning pale brown to brown when dry, loculate, locules elongate-ellipsoid, tubular to irregular in length, empty, filled with spores at maturity. ***Hymenium*** 18–43 μm thick. ***Basidia*** clavate, 2–3(–4)-spored, up to 8–10 μm over than hymenium, sterigmata 2–4 μm long, collapsed and disappeared at maturity.

**Figure 6.**
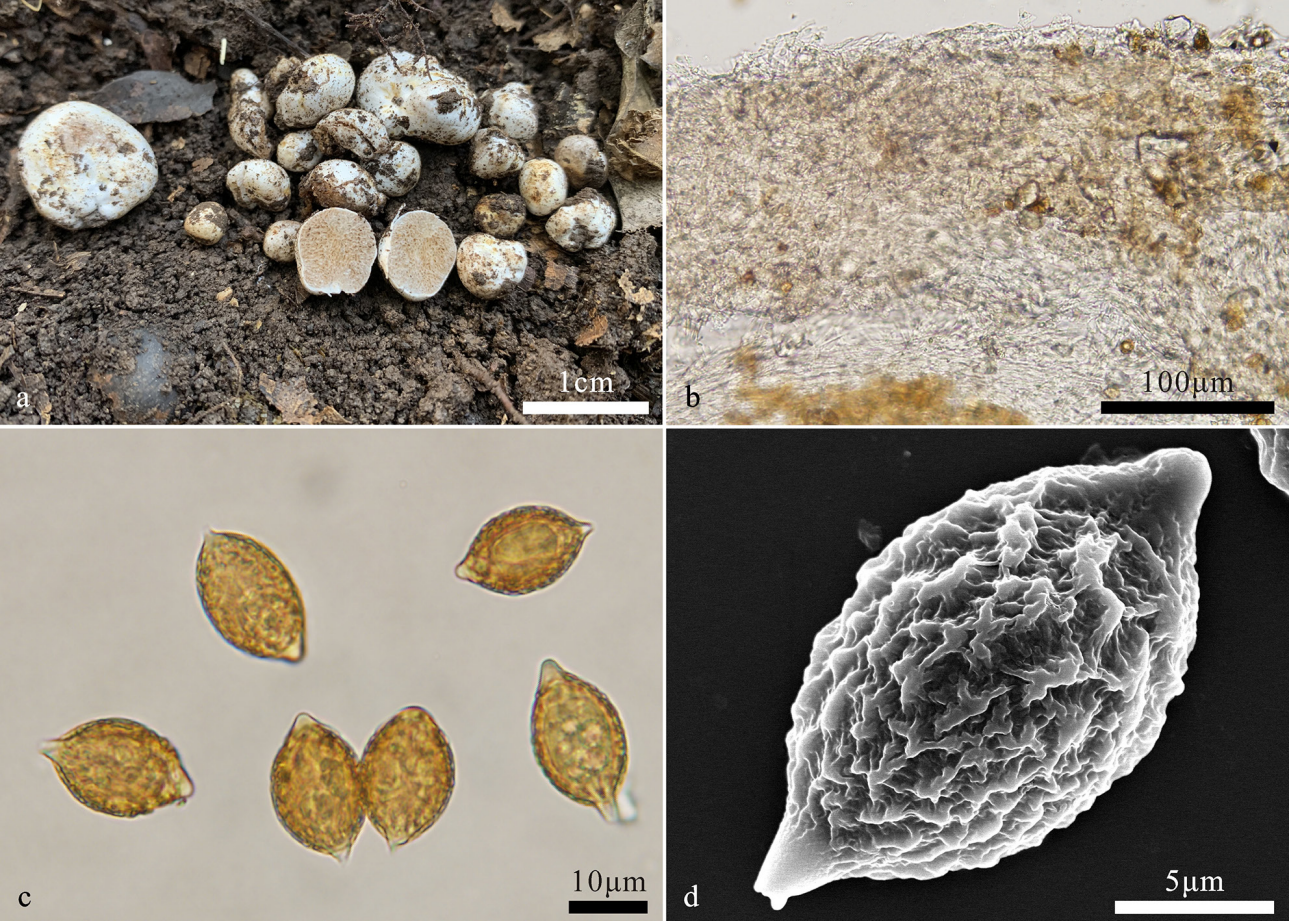
*Hymenogaster thermophilus* (BJTC FAN1269, holotype). **a**. Basidiomes; **b**. Peridium; **c**. Basidiospores; **d**. Basidiospore under SEM.

***Basidiospores*** broadly ellipsoid to ovoid, pale yellowish brown to yellowish brown at maturity, ornamented with densely verrucose and ridges of 0.5–1 μm high, ridges short, irregular, interwoven, (9.4–)11.2–16.7(–17.5) × (8.3–)8.9–12.2(–12.9) μm (Lm × Wm = 13.2 ± 1.2 × 10.5 ± 0.9 μm, n = 30), Q = 1.2–1.4 (Q_av_ = 1.3 ± 0.06, n = 30), gelatinous perisporium present, with a apical hump, obtuse, hyaline, 1–3 μm high, with a pronounced basal sterigma, truncate, 1–2 μm long.

##### Habit.

Hypogeous, in the soil under *Castanea mollissima*, *Quercus wutaishansea*, *Quercus* sp., and *Quercus variabilis*, Shanxi Province, northern China.

##### Additional specimens examined.

China • Shanxi Province, Linfen City, Xi County, Shenjiagou, alt. 1321 m, 10 September 2017, in soil under *Quercus wutaishansea*, HKB116 (BJTC FAN942); • ibid., Yuncheng City, Xia County, Taikuanhe, alt. 900 m, 27 October 2017, in soil under *Quercus* sp., LT064 (BJTC FAN1090); • ibid., HKB152 (BJTC FAN1114); • ibid., HKB153 (BJTC FAN1114); • ibid., alt. 970 m, in soil under *Quercus variabilis*, YXY128 (BJTC FAN1096); • ibid., alt. 856 m, 28 October 2017, YXY135 (BJTC FAN1122); • ibid., LT065 (BJTC FAN1125); • ibid., alt. 1057 m, 29 October 2017, in soil under *Castanea mollissima*, HKB166 (BJTC 1129); • ibid., alt. 970 m, 15 April 2017, YXY153 (BJTC FAN1159); • ibid., alt. 1350 m, 5 October 2020, LT142 (BJTC FAN1257); • ibid., 7 October 2020, LT150 (BJTC FAN1265); • ibid., LT154 (BJTC FAN1269); • ibid., LT155 (BJTC FAN1270). • Jincheng City, Qinshui County, Xiachuan Village, Shunwangping, alt. 1744 m, 30 October 2017, YXY140 (BJTC FAN1132); • Changzhi City, Qinyuan County, Lingkongshan Mountains, alt. 1521 m, LT074 (BJTC FAN1147).

##### Note.

*Hymenogaster thermophilus* is characterized by white to yellowish white basidiomes, broadly ellipsoid to ovoid with densely verrucose and ridge-like ornamentations, and relatively warmer habitats. BLASTn revealed that *H. tenuihymeninus* shares less than 95.5% ITS similarity with sequences from the NCBI database.

In our study, 12 ITS sequences retrieved from the NCBI database showed a match with *H. thermophilus*, indicating that the present species also occurs in Europe and North America, and is usually associated with trees of *Castanea*, *Fagus*, *Quercus*. Moreover, four of them labeled as *H. niveus* (Stielow et al. 2011), were actually misidentifications as *H. niveus* has basidiomes turning red when touched and relatively larger basidiospores (15–18.5 × 9.5–11.5 μm) (Stielow et al. 2011; Dodge and Zeller 1934).

#### Hymenogaster intermedius

Taxon classificationFungiAgaricalesHymenogastraceae

Stielow, Bratek & Hensel, PLoS ONE 6(1): e15614, 9 (2011)

D8C4F695-EEEF-54CA-935F-F754222D4C74

##### Habit.

Hypogeous, in the soil under *Pinus armandii*, Shanxi Province, northern China.

##### Specimens examined.

China • Shanxi Province, Yuncheng City, Yuanqu County, Lishan Mountain, alt. 2229 m, 17 October 2016, in soil under *Pinus armandii*, HKB038 (BJTC FAN673); • ibid., alt. 2218 m, 15 August 2016, HBD016 (BJTC FAN619); • ibid., YXY018 (BJTC FAN624); altitude 2209 m, in soil under *Pinus armandii*, 16 August 2016, HKB007 (BJTC FAN579), HKB008(BJTC FAN558), HKB009 (BJTC FAN559), HKB010 (BJTC FAN556), HKB013 (BJTC FAN582), HKB018(BJTC FAN552); • ibid., WYW009 (BJTC FAN562), WYW010 (BJTC FAN568), WYW011 (BJTC FAN577); • ibid., CM004 (BJTC FAN581); • ibid., altitude 2282 m, HBD006 (BJTC FAN594); • ibid., HKB005 (BJTC FAN612); • ibid., altitude 2229 m, 17 October 2016, in soil under *Pinus armandii*, SXY018 (BJTC FAN665).

##### Note.

The sequences from our 16 specimens well matched the type specimen of *H. intermedius* in the phylogeny (Fig. 1), indicating they are conspecific. This species is first reported in China and frequently encountered in Shanxi Province.

## Discussion

Shanxi Province in northern China encompasses a range of climates from subtropical to cold temperate. Prior to the study, 27 *Hymenogaster* species had been reported in Shanxi Province (Liu 1985, 1998; Liu et al. 1996; Tao et al. 1996; Li et al. 2024a, 2024b). Of these, eight species are supported by DNA evidence, i.e. *H. arenarius* Tul. & C. Tul., *H. citrinus* Vittad., *H. latisporus* L. Fan & Ting Li, *H. papilliformis* L. Fan & Ting Li, *H. perisporius* Ting Li & L. Fan, *H. pseudoniveus*, *H. variabilis* L. Fan & Ting Li, *H. zunhuaensis* (Li et al. 2024a, 2024b), while the remaining 19 species currently lack DNA data for the time being, i.e. *H. albus* (Bull.) Berk., *H. cangyanshanensis* Liu, *H. cerebellum* Cavara, *H. fragilis* Zeller & C.W. Dodge, *H. gilkeyae* Zeller & C.W. Dodge, *H. hessei* Soehner, *H. lycoperdineus* Vittad., *H. macrosporus* G. Cunn., *H. mischosporus* Soehner, *H. niveus* Vittad., *H. olivaceus* Vittad., *H. populetorum* Tul. & C. Tul., *H. reticulatus* Zeller & C.W. Dodge, *H. reticulatus* var. *laxisporus* K. Tao, Liu & Chang, *H. subglobosporus* K. Tao, Chang & Liu, *H. subnanus* K. Tao, Liu & Chang, *H. sulcatus* R. Hesse, *H. verrucofusisporus* Liu, K. Tao & Chang and *H. vulgaris* Tul. & C. Tul. (Liu 1985, 1998; Tao et al. 1996).

Due to the revocation of the herbarium (MHSU), almost all earlier specimens of *Hymenogaster* from Shanxi Province deposited in the herbarium got lost. So here we summarize the *Hymenogaster* species lacking DNA data only based on the original descriptions and newly collected specimens by the present authors.

Among the 19 species lacking DNA data, three species could probably be distinct, i.e. *H. mischosporus* (Liu 1998), *H. subnanus* and *H. verrucofusisporus* (Tao et al. 1996). Six species may be misidentified, including *H. gilkeyae*, *H. hessei*, *H. lycoperdineus*, *H. niveus*, *H. sulcatus*, *H. vulgaris*. Liu (1998) described the basidiospores of ‘*H. gilkeyae*’ ornamented with warts and short ridges and without utricle, which is completely different from that of *H. gilkeyae*. *Hymenogaster hessei*, *H. vulgaris* and *H. lycoperdineus* (Liu 1998) currently are synonyms of *H. griseus* (Stielow et al. 2011). *Hymenogaster griseus* is not confirmed from Shanxi Province in our analysis (this study). According to the descriptions by Liu (1998), the specimens under the names of *H. hessei* and *H. vulgaris* could probably be identical to *H. bulliardii* in dark and smooth basidiospores that seem unique to this species; the specimens under *H. lycoperdineus* have ridged ornaments on basidiospores, which does not fit *H. griseus*, whose basidiospores are warty or verrucose, and these specimens are likely identical to *H. pseudoniveus* (Li et al. 2024b), a frequently encountered species from Shanxi Province. *Hymenogaster niveus* and *H. sulcatus* (Dodge and Zeller 1934; Stielow et al. 2011) are not confirmed by our molecular evidence (Li et al. 2024a, 2024b; this study); however, *H. niveus* may be identical to *H. pseudoniveus* (Li et al. 2024b) or *H. albo-ovoideus* (this study), two newly established species based on local specimens; *H. sulcatus* is highly similar to *H. bulliardii*.

Two species are very similar to *H. intermedius* (Stielow et al. 2011; this study) in morphology, if not identical, namely *H. cangyanshanensis* (Liu 1985) and *H. cerebellum* (Liu 1998). Notably, a lectotype (HMAS81692 ex MHSU2320) based on a specimen from southern Shanxi Province designated for *H. cangyanshanensis* as its type specimen (MHSU 2320, Hebei Province) was destroyed in a fire. The two localities are situated in different climate zones and at a distance of more than 300 km, hence whether the lectotype is identical to the holotype is in question. Unfortunately, we were unable to sequence the DNA data successfully from the lectotype.

The remaining eight species have been transferred to other fungal genera or synonymized according to Index Fungorum, namely *H. albus*, *H. fragilis*, *H. macrosporus*, *H. olivaceus*, *H. populetorum*, *H. reticulatus*, *H. reticulatus* var. *laxisporus*, *H. subglobosporus*. *Hymenogaster albus* now is *Descolea alba* (Bull.) Kuhar, Nouhra & M.E. Sm.; *H. fragilis* has been transferred to *Cortinarius* as *C. fragilis* (Zeller & C.W. Dodge) Peintner & M.M. Moser; *H. subglobosporus* has broadly ellipsoid to subglobose basidiospores with incompletely reticulated ornamentation (Tao et al. 1996), *H. reticulatus*, currently its correct name is *Descolea reticulata* Kuhar, Nouhra & M.E. Sm., probably is the most similar to *H. subglobosporus*. In addition, the specimens under the names of *H. macrosporus*, *H. populetorum*, *H. reticulatus*, *H. reticulatus* var. *laxisporus* may be identical as all of them share the irregularly reticulate ornaments on basidiospores; *H. olivaceus* is a synonym of *H. citrinus* (Stielow et al. 2011). *Hymenogaster citrinus* has been confirmed from this province by molecular analysis (Li et al. 2024a).

Of the 14 species supported by DNA evidences and morphology in Shanxi Province (Li et al. 2024a, 2024b; this study), several species were found to exhibit exclusive host associations, i.e., *H. albovoideus* and *H. enuihymenium* are associated with *Betula costata* only; *H. latisporus* and *H. variabilis* with *Pinus tabulaeformis* only; *H. minisporus* with *Castanea mollissima* only; *H. pinophilus* and *H. intermedius* with *Pinus armandii* only; *H. subluteus* with *Quercus* spp. only. These host characteristics could have practical value for identifying these species. Climate types probably exert a significant influence on the distribution of *Hymenogaster* species across Shanxi Province. Most species thrive in warm climates. Specifically, those adapted to subtropical environments, such as *H. albovoideus*, *H. intermedius*, *H. latisporus*, *H. perisporius*, *H. subluteus*, *H. thermophilus*, *H. variabilis*, and *H. zunhuaensis*, are restricted to the southern regions. In contrast, only two species, *H. arenarius* and *H. tenuihymenium*, are found in the cold temperate climate of subalpine areas in the north. The distribution of *H. citrinus* and *H. papilliformis* spans both central and northern regions, whereas *H. pseudoniveus* is the only species observed across all climate types in the province.

Moreover, some *Hymenogaster* species have been reported from other regions of China excluding Shanxi Province, and Asian countries. These species are: *H. indicus* D.P. Tiwari, Harsch & B.K. Rai, *H. kwangsiensis* Liu, *H. latifusisporus* K. Tao, Chang & Liu, *H. minisporus* T. Li & L. Fan, *H. ozeensis* Kobayasi, *H. pacificus* Kobayasi, *H. verrucosus* Bucholtz, *H. vittatus* Chang, K. Tao & Liu, *H. xizangensis* K. Tao, Liu & A. S. Xu (Dodge and Zeller 1934; Kobayasi 1937, 1979; Tiwari et al. 1991; Liu 1980; Tao et al. 1996). Among these, *H. minisporus* is supported by DNA evidence, while the remaining eight species lack molecular data. These species can still be distinguished from our new species based on their unique morphology and habitat.

Specifically, *Hymenogaster indicus* is associated with *Shorea robusta* (Tiwari et al. 1991); *H. kwangsiensis*, previously transferred to *Timgrovea* Bougher & Castellano (Bougher and Castellano 1993), and *H. latifusisporus* both possess basidiospores with reticulate verrucose ornamentation. *H. kwangsiensis* has ovoid to broadly ellipsoid basidiospores, whereas *H. latifusisporus* has ellipsoid basidiospores (Liu 1980; Tao et al. 1996); *H. verrucosus* and *H. vittatus* both feature fusiform to ellipsoid basidiospores, with *H. verrucosus* ornamented with irregularly rugose-sinuose (Dodge and Zeller 1934), and *H. vittatus* ornamented with sparse reticulate (Tao et al. 1996); *H. ozeensis* and *H. pacificus*, both from Japan, have smooth basidiospores, with *H. ozeensis* having smaller spores (8–11 × 3–4 μm) (Kobayasi 1937, 1979); *H. xizangensis* has fusiform-ellipsoid basidiospores (10–13 × 7.5–8.5 μm), ornamented with irregularly verrucose (Tao et al. 1996).

## Supplementary Material

XML Treatment for Hymenogaster albo-ovoideus

XML Treatment for Hymenogaster pinophilus

XML Treatment for Hymenogaster subluteus

XML Treatment for Hymenogaster tenuihymeninus

XML Treatment for Hymenogaster thermophilus

XML Treatment for Hymenogaster intermedius

## Additional information

### Artificial Intelligence (AI) use

The authors accept full responsibility for the content of the manuscript, including the disclosure of any use of AI.

No AI tools were used in the preparation of this manuscript.

### Author ORCIDs

N. Mao https://orcid.org/0000-0003-1564-9446

T. Li https://orcid.org/0000-0001-7809-3217

H.-Y. Fu https://orcid.org/0000-0001-5529-4964

Y.-X. Zhang https://orcid.org/0000-0002-6757-1903

Y.-J. Zhang https://orcid.org/0000-0002-6331-2189

L. Fan https://orcid.org/0000-0001-9887-7086

### Data availability

All of the data that support the findings of this study are available in the main text.
